# A kinematic, imaging and electromyography dataset for human muscular manipulability index prediction

**DOI:** 10.1038/s41597-023-02031-3

**Published:** 2023-03-11

**Authors:** Óscar G. Hernández, Jose M. Lopez-Castellanos, Carlos A. Jara, Gabriel J. Garcia, Andres Ubeda, Vicente Morell-Gimenez, Francisco Gomez-Donoso

**Affiliations:** 1 Human Robotics group, University of Alicante, 03690 San Vicente del Raspeig, Alicante, Spain; 2 Institute for Computing Research, P.O. Box 99, 03080 Alicante, Spain

**Keywords:** Health occupations, Computer science

## Abstract

Human Muscular Manipulability is a metric that measures the comfort of an specific pose and it can be used for a variety of applications related to healthcare. For this reason, we introduce KIMHu: a Kinematic, Imaging and electroMyography dataset for Human muscular manipulability index prediction. The dataset is comprised of images, depth maps, skeleton tracking data, electromyography recordings and 3 different Human Muscular Manipulability indexes of 20 participants performing different physical exercises with their arm. The methodology followed to acquire and process the data is also presented for future replication. A specific analysis framework for Human Muscular Manipulability is proposed in order to provide benchmarking tools based on this dataset.

## Background & Summary

Being able to estimate the Human Muscular Manipulability (HMM) index is very useful for a range of different tasks. HMM provides a measure that describes the relationship of an articulated body with respect to velocity, acceleration or force. As stated in^1–5^, the HMM index can be used to evaluate the effects of instantaneous variations between joints and limbs, and it is usually represented by a spheroid around the endpoint of the joint mechanism. The dimensions of this spheroid around the endpoint represent the maximal feasible velocity, acceleration or force capacity in the spheroid axes directions, giving a quantitative measure to evaluate the ability in the manipulation task. If this analysis is applied to a human limb, HMM is correlated with the comfort of an specific pose in order to generate movements at the end of the arm. For instance, HMM can be used to track a certain physical rehabilitation procedure after an injury under the assumption that the movements of the user are limited. Thus, if a person has an injured elbow that limits their movement and they need to grasp an object, they will try to compensate the reduced mobility by straining the shoulder and the wrist excessively. HMM in the natural set of poses to grasp an object would be lower than in the aforementioned case where shoulder and wrist are heavily stressed. In wearable robotics, control algorithms can thus be tuned to decrease unnecessary physical effort and for that reason, HMM can be used to improve the control input through its assessment in exoskeletons for assistance of disabled people^3^ or for power augmentation^6^, and in rehabilitation therapies with collaborative robots arms^7^. Thus, the ability to estimate HMM from a human pose has several applications to rehabilitation and healthcare, telemedicine, physiotherapy and labour risk prevention. Moreover, human motion is ultimately commanded by the electrical activation of muscles. To measure this activity, it is possible to use non-invasive Electromyography (EMG). Muscular information is directly related to postural outcomes during the performance of upper-limb movements, so it is expected that factors such as the aforementioned HMM could be predicted or inferred from EMG recordings. In this context, previous approaches on neuromechanical modelling have explored the possibility of extracting motion kinematics and dynamics from EMG signals in a variety of tasks^8,9^.

The bloom recently experienced by the new trends in artificial intelligence methods, and specifically machine learning and deep learning algorithms, has led to more accurate and efficient data processing of EMG data. For instance, some popular deep learning-based approaches consider the temporal factor using Long Term Short Memory units^10^. It is also popular to model the problem so it is based on images that can be efficiently processed by a convolutional pipeline^11,12^. Some other approaches leverage a fusion of features under a deep learning framework^13,14^. Nonetheless, traditional machine learning algorithms applied to control^15^ and neuromuscular modelling^16^ also reportedly provide good results.

Thus, the most common and most accurate approaches to perform predictions are based in machine learning and deep learning methodologies, which are notorious for being data-hungry algorithms. In this sense, in order to train these systems, datasets should provide large amounts of annotated data. In this work, we propose KIMHu, a Kinematic, Imaging and electroMyography dataset for HMM prediction. Samples are composed of images, depth maps, a list of keypoints that define the 3D position of the joints in the human body, and EMG signals. The labels include HMM indexes such as Kinematic Manipulability Index (KMI), Dynamic Manipulability Index (DMI), and a custom metric named Local Conditioning Index (LCI). The dataset features a number of physical upper-limb exercises, different repetitions of the same exercise, and data recorded from 20 healthy participants. In addition, benchmarking tools based on this dataset are introduced as well. Different train and test methodologies are suggested alongside accuracy metrics to be measured. Finally, a basic baseline has been set for comparison purposes.

### Similar datasets

Recent neuromechanical datasets, i.e., those which include both EMG and kinematic and/or kinetic information, are mainly divided into gait analysis and upper-limb activities. Gait datasets usually include 3D ground force reactions, MoCap-based kinematic estimation and EMG recordings on the leg muscles^17–19^. Another recent dataset includes also ultrasound imaging to infer neuromuscular information of dynamic gait^20^. Some of them do not include muscular activity^21^, missing the neuromechanical approach for further analysis. Regarding upper-limb activities, the variability of related datasets is higher. A variety of contributions are centered on only hand movements^22–25^. To our knowledge, only a previous dataset focuses on elbow movements, but only measuring isometric muscle contractions^26^. Several of the previously cited datasets also include high-density EMG on the forearm^22,26^. Our dataset includes not only HMM indexes but raw RGB image frames, depth maps and EMG information. Table 1 summarizes the main differences of all these datasets in comparison to our proposal.

**Table 1:** Summary of recent neuromechanical datasets vs Ours.

Dataset	P	LOC	KIN	EMG			RGB	D	M	F
				C	S	HD				
OURS	20	Arm	3D Cam	4	✓	✗	✓	✓	24	✗
Schreiber, 2019 ^17^	50	Legs	MoCap	8	✓	✗	✗	✗	52	✓
Matran-Fernandez, 2019 ^22^	25	Hand	Glove	8	✓	✗	✗	✗	18	✗
Jarque-Bou, 2019 ^23^	22	Hand	Glove	8	✓	✗	✗	✗	18	✗
Lencioni, 2019^18^	50	Legs	MoCap	8	✓	✗	✗	✗	37	✓
Rojas-Martínez, 2020^26^	12	Elbow	—	384	✓	✓	✗	✗	—	✓
Maleševic, 2021^24^	20	Hand	—	128	✓	✓	✗	✗	—	✓
Moreira, 2021^19^	16	Legs	MoCap	8	✓	✗	✗	✗	24	✓
Reznick, 2021^21^	10	Legs	MoCap	—	—	—	✗	✗	28	✓
Furmanek, 2021^25^	10	Hand	MoCap	10	✓	✗	✗	✗	3	✗
Zhang, 2022^20^	5	Legs	—	8	✓	✗	✗	✗	—	✓

P - Participants (all healthy); LOC - Location on the body; KIN - Method to extract kinematics; EMG - C: channels, S: sEMG, HD: high-density; RGB - Image frames; D: Depth Maps; M - number of markers; F - force recordings; HMM - Human Manipulability Indexes (LMI, DMI and LCI).

## Methods

In this section, the methodology followed to generate the data is explained. In addition, a description of the capture setup and the techniques applied to synchronize and generate the ground truth are presented.

### Overview

The KIMHu dataset is a collection of data comprised of images, depthmaps, skeleton tracking, electromyography data of the upper limbs and different Human Muscular Manipulability indexes such as Kinematic Manipulability Index, Dynamic Manipulability Index and Local Conditioning Index. It depicts different participants and different upper limbs physical exercises. The proposed dateset could be used, but not limited, to:

- Prediction of Human Muscular Manipulability based on image data.
- Prediction of Human Muscular Manipulability based on 3D data or depth maps.
- Prediction of Human Muscular Manipulability based on EMG data.

Ultimately, it could help developing different applications. For instance, the HMM indexes could be used for a physical rehab measurement software, or occupational hazard monitoring applications. Finally, the methodology proposed can be replicated by the scientific community in order to include more movements, more participants or data of different nature.

### Capture setup

In this Section, detailed information about the devices used to record the data is introduced. The corresponding software specifications are described as well.

#### Hardware

**Fig. 1:**
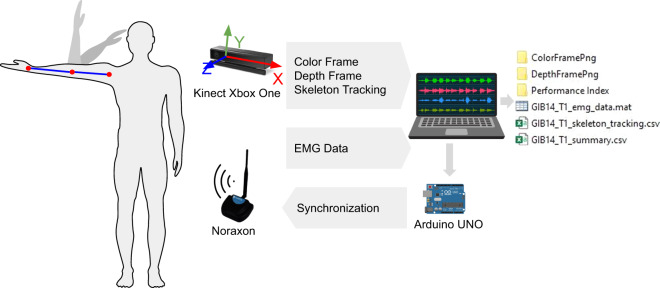
Capture Setup.

The architecture of the system is composed of a RGB-D camera (Microsoft Kinect Xbox One, also known as Kinect V2), a Noraxon’s TeleMyo Mini DTS system, an Arduino UNO and a laptop with an Intel Core i7-9750H 2.60 GHz processor and 16GB RAM. The proposed system is shown in Fig. 1.

The second-generation Kinect V2 by Microsoft (released in July, 2014) is equipped with a color camera, depth sensor (including infrared (IR) camera and IR projector) and a microphone array. The Kinect V2 can be used to capture color images, user skeleton joint tracking, depth images, IR images and audio information^27^. The detailed technical specifications are summarized in Table 2.

**Table 2:** Kinect V2 hardware specifications.

RGB camera (pixel)	1920 × 1080
Depth camera (pixel)	512 × 424
Max depth distance (m)	4.5
Min depth distance (m)	0.5
Horizontal FOV (degrees)	70
Vertical FOV (degrees)	60
Tilt motor	No
Skeleton joints define	25
Full skeleton tracking	6
USB	3.0

Regarding to the mini DTS system, this is composed of a mini DTS receiver and four single channel DTS EMG wireless sensors with a sample rate of 1500 Hz. The measured data of each sensor is transmitted directly to the receiver in a short-range network with radio frequencies transmissions between 2.4 and 2.5 GHz. The EMG sensor data acquisition system has a 16-bit resolution and the EMG sensors have an input range of 5 mV and a first order high-pass filter set to 10 2 Hz is applied internally by the hardware system, the baseline noise of the sensors is lower than 5 V RMS of the signal. More technical details are included in Table 3.

**Table 3:** Noraxon Mini DTS hardware specifications.

Sensor transmission range	20 meter
Selectable low-pass cutoff	500/1000/1500 Hz
Wireless update rate	100 Hz
Selectable sample rate	3000/1500 Hz
Differential Input impedance	> 10 Mohm
Baseline noise	5 uV RMS
Electronic Gain	200
Overall Gain	500

#### Software

The implemented algorithm to capture Kinect information is written in C# and uses Microsoft Kinect SDK 2.0 on Windows 11 operating system. The Microsoft Kinect SDK 2.0 can extract skeleton data at approximately 30 Frames Per Second (FPS), the source code for capturing and processing the information is available at^28^. EMG signals were captured with Noraxon’s Myo Muscle software and exported into Matlab compatible files.

### Synchronization

To capture information, a Kinect V2 (30 Hz) and a Noraxon Mini DTS (1500 Hz) are used. A digital pulse is sent to the Noraxon via the arduino UNO to indicate the beginning and the ending of each test. The connection between the devices is shown in Fig. 1.

### Procedure

Twenty participants were included in the study: three women and seventeen men, age = 27±8 years, height = 175±6 cm, weight = 75±14 Kg, as presented in Table 4. All participants were right-handed and had no known neuromuscular or sensory disorders. Prior to their participation, the participants were informed of the study approach and gave their informed consent in accordance with the code of ethical conduct. The study was conducted in accordance with the Declaration of Helsinki, and approved by the Ethics Committee of University of Alicante (protocol code UA-2021-06-21-01, approved on 29 June 2021.

**Table 4:** Anthropometric information of the participants.

Participant ID	Gender	Age	Height (m)	Weight (Kg)	Upper arm length(m)	Forearm length(m)	Hand length(m)
EXT13	F	22	1.69	69	0.238	0.218	0.125
EXT14	M	31	1.71	90	0.248	0.239	0.153
EXT15	M	49	1.73	90	0.255	0.230	0.128
EXT16	M	40	1.75	92	0.248	0.235	0.154
EXT18	F	29	1.67	62	0.251	0.220	0.156
EXT19	M	28	1.72	75	0.257	0.234	0.145
EXT20	M	21	1.71	70	0.244	0.227	0.147
GIB13	M	20	1.73	64	0.236	0.216	0.154
GIB14	M	21	1.73	65	0.248	0.230	0.161
GIB15	M	22	1.82	78	0.258	0.245	0.162
GIB16	M	21	1.75	70	0.244	0.236	0.152
GIB17	M	20	1.80	55	0.269	0.238	0.159
GIB18	F	20	1.73	63	0.247	0.222	0.144
GIR26	M	21	1.75	70	0.257	0.246	0.160
GIR27	M	22	1.85	105	0.258	0.248	0.160
GIR28	M	21	1.93	100	0.267	0.275	0.185
I03	M	34	1.78	91	0.263	0.236	0.174
I04	M	33	1.64	70	0.252	0.228	0.154
I05	M	31	1.73	70	0.249	0.235	0.151
MAYR02	M	24	1.70	57	0.247	0.225	0.140

Prior the execution of the exercises, the participants were asked to stand over two markings on the floor, in front of the RGBD camera, in a resting position. The user must perform two tests. In each test, the participant must execute ten repetitions of the proposed exercise, having a resting period of six seconds between each repetition. In front of the participants a visual guidance was displayed on a smartphone to indicate the resting period and the exercise execution period. In total, both exercises took about 15 minutes.

**Fig. 2:**
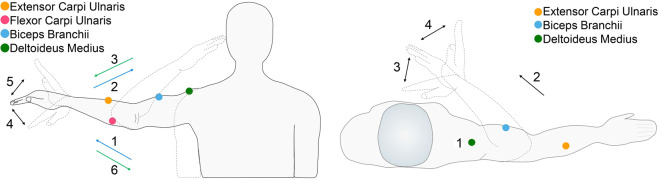
Tests: Leftmost image shows the Movements performed in Test 1 (Frontal plane) and the rightmost depicts the movements performed in Test 2 (Transverse plane).

For the first exercise, the participants must extend their arm, i.e., to do an abduction movement with the forearm in pronation. After that, an elbow-flexion towards the head must be executed and then, the participants must return to the elbow-extension position. Sequentially, the participants must execute a wrist-flexion movement, return the wrist to a neutral position, and then perform a wrist-extension movement. Finally, the participants must take their arm to the resting position. In Fig. 2 the sequence of movements required is depicted.

For the second exercise, the participants must execute an abduction of the arm and then, execute an elbow-flexion movement and place their wrist right in front of their chest. After that, execute a wrist-flexion movement, then return the hand to a neutral position of the wrist and subsequently, execute a wrist-extension movement. At the end, the participants must take their arm to the resting position. In Fig. 2 some steps of the exercise can be observed.

## Data Records

In this Section, the data that KIMHu^29^ provides is explained in detail, including the EMG data, the skeleton tracking, the images and the HMM metrics. It is worth noting that the KIMHu dataset can be downloaded from ScienceDB 10.57760/sciencedb.01902.

### EMG Data

During the execution of the exercises, the surface EMG signals were recorded using bipolar electrodes over the following muscles: Extensor Carpi Ulnaris, Flexor Carpi Ulnaris, Biceps Branchii and Deltoideus Medius.

### Images and user skeleton joint tracking

**Fig. 3:**
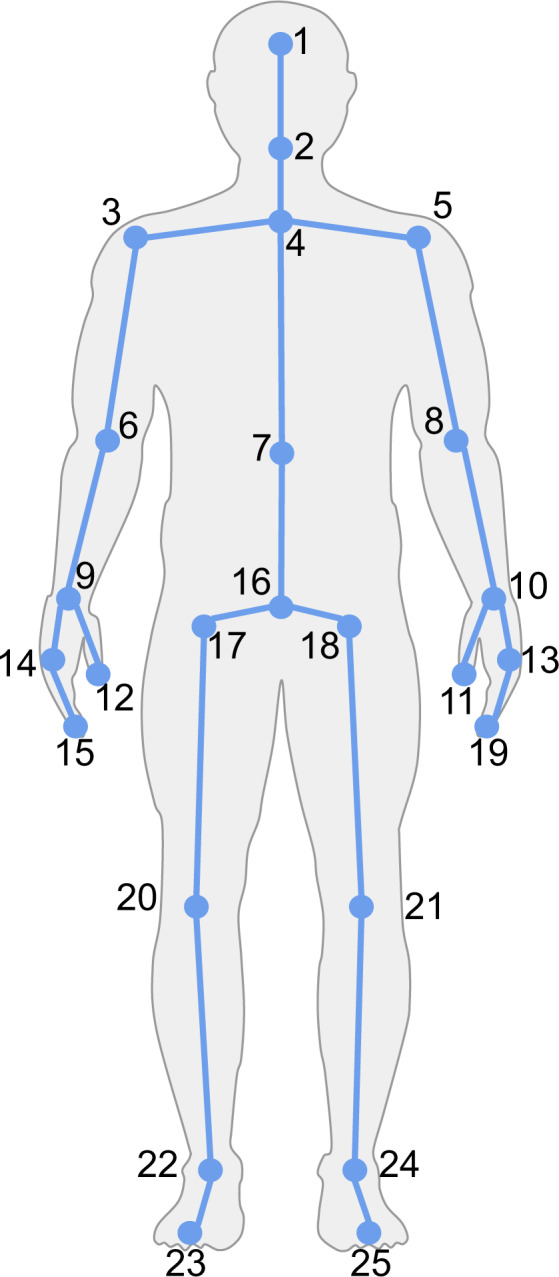
Body tracking joints.

During the execution of the exercise routine, features of the participant’s posture are extracted for each frame. These features consist in the three dimensional (3D) points representing the position of each one of the skeleton joints. This information must be conveniently parsed and stored so that representative features can be easily extracted. CSV files are employed to store the position and orientation of the 25 joints comprising the skeleton (see Fig. 3). Moreover, additional information such as the timestamp is also recorded. The depth maps are recorded by the IR camera, they are 2D images 16 bits encoded in which measurement information is stored for each pixel. Color images are captured by the second lens of the RGBD camera. The names of the files have the following nomenclature: color <timestamp>.png and depth <timestamp>.png respectively.

### Kinematic groud truth

In the following subsections, the different HMM indexes included in the dataset are explained, focusing on what they mean and how they were computed.

### Human arm kinematic model

**Fig. 4:**
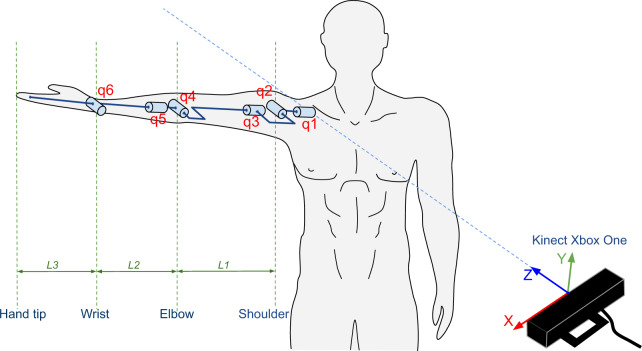
Human Arm Kinematic Model.

To compute the measure of muscle manipulability, the musculoskeletal system of the human right arm is modeled as a three-segment mechanism (see Fig. 4), where the first joint is the shoulder, the second joint is the elbow, and the third joint is the wrist. This model has six degrees of freedom: shoulder abduction/adduction (q1), shoulder flexion/extension (q2), shoulder rotation (q3), elbow extension/flexion (q4), elbow pronation/supination (q5), and wrist flexion/extension (q6). L1, L2, and L3 represent the lengths of the arm, forearm, and hand, respectively.

### Manipulability

**Fig. 5:**
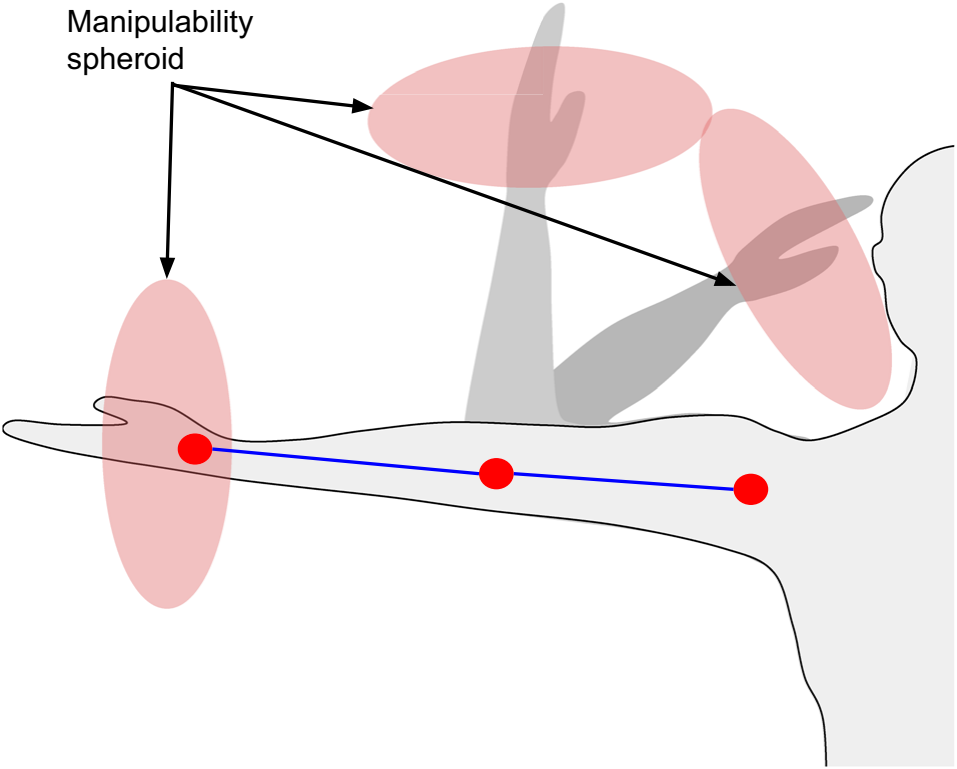
Example of three different arm positions and their corresponding muscular force manipulability ellipses.

HMM is a kinematic concept to compute the dexterous manipulability of a joint mechanism (as the human arm). Quantitatively, HMM is a measure that describes the relationship between joints and limb endpoint with respect to velocity, acceleration or force that can be applied by the mechanism to perform manipulation tasks. This concept can be represented by a spheroid around the endpoint to represent the maximal feasible velocity, acceleration or force capacity in the spheroid axes directions (see Fig. 5). Yoshikawa^30^ defined this paramenter of manipulability for a joint manipulator with a scalar value given by:

1 $$\omega =\sqrt{det\left[J{J}^{T}\right]}$$

Where J is the 6xN Jacobian matrix of the manipulator in a specific position, where N is the number of joints of the manipulator. Yoshikawa also defined the concept of dynamic manipulability^31^, where the dynamics of the joint manipulator is taken into account when determining its manipulability scalar value:

2 $${\omega }_{d}=\sqrt{det\left[J{\left({M}^{T}M\right)}^{-1}{J}^{T}\right]}$$

Where M is the inertia matrix.

**Fig. 6:**
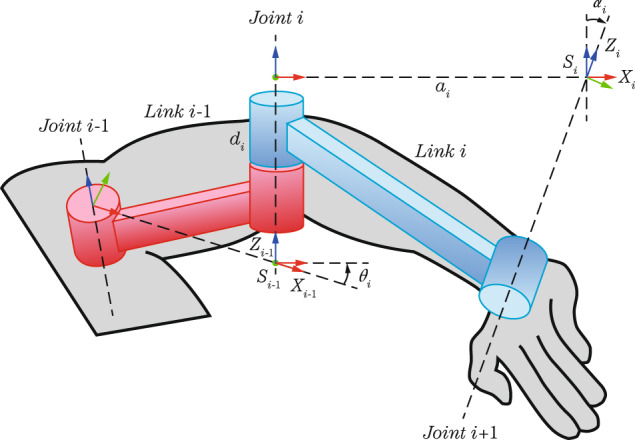
Visualization of the DH parameters and the DH reference systems.

For this approach, the human arm was modelled in Matlab by means of the Denavit Hartenberg (DH) parameters^32^ and using a robotics toolbox^33^. The DH representation for the right arm is given on the Table 5. In each row of this table, it can be seen the four DH parameters *a*_*i*_, *α*_*i*_, *d*_*i*_ and *θ*_*i*_, which describe the transformations between the DH reference systems *S*_*i-*1_ and *S*_*i*_, located in the joints of the upper arm (see Fig. 6). The six rows of Table 5 define the whole kinematics of the arm and it allows to compute the position of the endpoint from given joint values (q1,…,q6).

**Table 5:** DH table for 6-DOF human arm.

i	*θ*_*i*_	*α*_*i*_	*a*_*i*_	*d*_*i*_
1	q1	$\frac{\pi }{2}$	0	0
2	q3	$\frac{\pi }{2}$	0	0
3	q3	$\frac{\pi }{2}$	0	−*L*_1_
4	q4	$-\frac{\pi }{2}$	0	0
5	q5	$-\frac{\pi }{2}$	0	*L*_2_
6	q6	0	*L*_3_	0

The angles for each of the degrees of freedom, as well as the lengths of the links, are computed using the points captured by the RGBD camera: shoulder, elbow, wrist, thumb and hand tip. With the configuration of the arm, the information on the angles and the inertia matrix, the kinematic and dynamic manipulability is computed by applying equations?? and?? respectively.

### Local conditioning index

There are other parameters which provides a good and intuitive measure about the dexterity of the joint mechanism when it comes to grasp or manipulate an object. They are the Dexterity Index (*I*_*d*_), which provides a measure of how comfortable the joint mechanism is working; and the Local Conditioning Index (*I*_*d*_), which gives a value between 0 and 1, the closer to 1 the system has better dexterity, and the closer to 0, the joint mechanism is closer to a singularity^34^.

3 $${I}_{d}\left(J\right)=\left\Vert J\right\Vert \cdot \left\Vert {J}^{-1}\right\Vert $$

4 $${I}_{cl}={I}_{d}{\left(J\right)}^{-1}$$

### Organization

Upon downloading, 40 folders can be found in the root of the dataset. Each folder corresponds with one participant, whose identifiers are defined in Table 4, followed by T01 or T02, being the numbers the identifier of the movement as shown in Figure 2. Thus, each folder contains the data of a movement of a single parcitpant. Inside each folder, there are the following folders and files:

- ColorFramePng (Folder): Contains the color frames corresponding with the user and test.
- DepthFramePng (Folder): Contains the depth maps frames corresponding with the user and test.
- Performance Index (Folder): Contains a csv file with the KMI, DMI and LCI, and a pdf of the data plotted for visualization.
- XX_YY_emg_data.mat (File): Contains the EMG data corresponding with the user and test.
- XX_YY_skeleton_tracking (File): Contains the keypoint tracking data corresponding with the user and test.
- XX_YY_summary (File): Is a summary of the gathered data for each timestamp.

## Technical Validation

The amount of data (number of files and disk space) categorized by test is shown in Table 6. In total, the KIMHu dataset comprises 5400304 synchronized samples for each color image, depth map and skeleton data. The same number for each channel of the EMG stream and HMM indexes are also included. There were twenty participants involved in this study with different anthropometric features to ensure a proper coverage of the sample space.

**Table 6:** Dataset metrics for each test.

Metric	Color Image Count	Depth Map Count	Skeleton Estimation Count	Total
Test Code				
T01	90053			270159
T02	89957			269871
**Total**	180010			540030
**Disc Space (GB)**	257.61	28.20	1.16	286.97

### Benchmark

One of the goals of our dataset is to serve as a benchmark and a global framework of comparison for HMM prediction systems. Thus, alongside the data itself, a set of methodologies to enable an easy and fair comparison are also proposed. These methodologies, which are thoroughly described in this section, include different strategies to split data into train and test and how to compute accuracy metrics. Finally, a baseline approach that takes EMG signals as input and predicts the corresponding KMI, DMI and LCI is proposed as well.

### Train and test splitting methodologies

In order to properly test the learning algorithms and the generalization capabilities, two different train and test splitting methods are proposed.

First, all the samples must be shuffled together. A sample is understood as a piece of data that is correlated to an expected output, so it depends on the approach. For instance, it could be a system that takes as input a number of EMG data to predict the corresponding KMI. In this case, a sample is composed of *time_steps* × *emg_channels*. Thus, after the samples are randomized, 70% should be used for training and the remaining 30% for testing the algorithms. This methodology, which follows default figures in machine learning, ensures that the algorithm has enough data to be properly trained whilst it is tested against unknown data. Both splits ensure the presence of samples of all the range of input and output data however do not provide insight about the generalization capabilities towards different human participants as all of them are present in both the training and the test set. This is inconvenient as the algorithm could perform unexpectedly when deployed in a real use case where the participants were not considered in the training process. Nonetheless, it still is a valuable metric to measure the performance of the approach.

Due to this, a second splitting method is suggested. In this case, the samples of random 14 participants should be used for training, and the remaining 6 for testing. By training and testing with a set of different users, a proper measure of the generalization capabilities of the methods can be obtained, thus providing insight about the real performance on an actual use case.

For the sake of comparison, these methodologies are named “data-centric” and “user-centric” respectively. Finally, it is worth mentioning that all metrics should be reported for both splitting methods.

### Metrics and comparison framework

As stated before, in order to set a proper comparison framework for the scientific community, the metrics that should be reported are proposed here.

At this point, it is worth recalling that the labels of the dataset KMI, DMI and LCI are real and continuous values, thus the regular categorical accuracy is not meaningful.

This is why the computation of the percentage of test samples that yield an error below different thresholds should be reported. These measures provide an easy-to-understand score about the performance of regression models. The thresholds for KMI are *T*01 = 0.0001, *T*1 = 0.001 and *T*2 = 0.002. The thresholds for DMI are *T*01 = 0.05, *T*1 = 0.5 and *T*2 = 1. The thresholds for LCI are *T*01 = 0.001, *T*1 = 0.01 and *T*2 = 0.02. These values are set to provide three difficulty levels, from most restrictive to laxer. For instance, the KMI in the dataset varies from 0 to ~0.02, so an error of *T*01 represents a displacement of a 0.5% regarding the total range of values.

The error that should be obtained for each sample is the Absolute Error (AE), which is defined as the absolute difference between the actual value of the label *y* and the predicted value *f*(*x*), that is $$AE=| y-f(x)| $$.

### Baseline

In order to set a basic baseline, a range of state-of-the-art regression methods were adopted to predict the HMM values from EMG input. To train the algorithms, the proposed dataset was processed using the sliding window method with a stride of 5. This is done to provide the algorithms with temporal context. Each sample is composed of 512 contiguous readings of the 4 EMG sensors, so each sample has 2048 features. The corresponding label is the KMI, DMI or LCI of the last instant considered in the sample. Both data-centric and user-centric splitting methodologies were followed, as defined in Section Train and Test Splitting Methodologies. Different models were trained regarding the 3 different HMM problems mentioned before for each regression algorithm. Finally, the obtained results are shown in Table 7. The values reported were computed as explained in Section Metrics and Comparison Framework.

**Table 7:** Metrics obtained for prediction of the KMI, DMI and LCI from EMG data by different regression approaches to serve as a baseline.

Data-centric splitting method									
Regressor	KMI			DMI			LCI		
	T01	T1	T2	T01	T1	T2	T01	T1	T2
LightGBM	0.98	10.05	23.33	5.58	52.22	84.26	4.60	48.89	91.06
Linear Reg.	0.73	7.61	16.07	4.36	43.18	77.95	2.08	23.78	89.19
XGBoost	1.18	12.05	25.81	6.18	54.96	84.36	6.59	52.97	89.23
ElasticNet	0.71	7.33	15.45	4.31	42.80	79.37	1.98	22.23	93.03
User-centric splitting method									
LightGBM	0.77	7.9	23.11	5.48	50.65	89.96	2.68	56.83	94.15
Linear Reg.	0.62	6.26	13.45	3.61	36.64	80.70	1.69	19.77	89.70
XGBoost	1.12	11.80	27.13	5.08	50.54	86.03	6.84	59.21	90.50
ElasticNet	0.59	5.92	12.80	3.96	33.91	82.35	1.51	17.94	92.12

Values are percentages regarding the number of samples in the test split, so a perfect score would be 100.

First, LightGBM^35^ was adopted. This is a framework, created by Microsoft, that provides a special implementation of a Gradient Boosting Decision Tree classifier. Particularly, LightGBM introduces two main features. First, gradient-based one-side sampling discards input data instances with small gradients, significantly reducing the number of samples. On the other hand, exclusive feature bundling enables the bundling of mutually exclusive features reducing, thus, its number. Both improvements allows LightGBM to provide accurate predictions whilst keeping the computation cost at bay. This method was selected among other because it has reportedly shown comparable performance to deep-learning algorithms in certain problems, and has been successfully used for a range of tasks such as land cover classification^36^, regression of structure-activity relationships^37^ or delivery time prediction^38^.

Linear Regressor was also employed. This is an ordinary least squares linear regression. This algorithm fits a linear model with coefficients $w=({w}_{1},...,{w}_{p})$ to minimize the residual sum of squares between the observed targets in the dataset, and the targets predicted by the linear approximation. This is a naive but useful regression algorithm.

XGBoost was also involved in the benchmark. XGBoost stands for “Extreme Gradient Boosting”, which is heavily based in^39^. XGBoost works as Newton-Raphson in function space unlike gradient boosting that works as gradient descent in function space, a second order Taylor approximation is used in the loss function to make the connection to Newton Raphson method.

Finally, the last algorithm is ElasticNet. The main purpose of ElasticNet regression is to find the coefficients that minimize the sum of error squares by applying a penalty to these coefficients. ElasticNet combines L1 and L2 (Lasso and Ridge) approaches. As a result, it performs a more efficient smoothing process. Elastic Net first emerged as a result of critique on Lasso, whose variable selection can be too dependent on data and thus unstable. The solution proposed by the algorithm is to combine the penalties of Ridge regression and Lasso to get the best of both worlds.

## Data Availability

Examples are provided for information processing with this dataset, this code is freely available in the following repository: https://github.com/VicenteMorell/KIMHu.

## References

[1] Tanaka, Y., Yamada, N., Nishikawa, K., Masamori, I. & Tsuji, T. Manipulability analysis of human arm movements during the operation of a variable-impedance controlled robot. In *2005 IEEE/RSJ International Conference on Intelligent Robots and Systems*, 1893–1898, 10.1109/IROS.2005.1545519 (2005).

[2] Ohta K, Tanaka Y, Kawate I, Tsuji T (2014). Human muscular mobility ellipsoid: End-point acceleration manipulability measure in fast motion of human upper arm. Journal of Biomechanical Science and Engineering.

[3] Petrič, T., Peternel, L., Morimoto, J. & Babič, J. Assistive arm-exoskeleton control based on human muscular manipulability. *Frontiers in Neurorobotics* **13**, 10.3389/fnbot.2019.00030 (2019). 10.3389/fnbot.2019.00030 PMC6548979 31191289

[4] Jacquier-Bret J, Gorce P, Rezzoug N (2012). The manipulability: a new index for quantifying movement capacities of upper extremity. Ergonomics.

[5] Tanaka Y, Nishikawa K, Yamada N, Tsuji T (2015). Analysis of operational comfort in manual tasks using human force manipulability measure. IEEE Transactions on Haptics.

[6] Goljat, R., Babič, J., Petrič, T., Peternel, L. & Morimoto, J. Power-augmentation control approach for arm exoskeleton based on human muscular manipulability. In *2017 IEEE International Conference on Robotics and Automation (ICRA)*, 5929–5934, 10.1109/ICRA.2017.7989698 (2017).

[7] Chiriatti G, Bottiglione A, Palmieri G (2022). Manipulability optimization of a rehabilitative collaborative robotic system. Machines.

[8] Saxby D (2022). Machine learning methods to support personalized neuromusculoskeletal modeling. Biomechanics and Modeling in Mechanobiology.

[9] Durandau G, Farina D, Sartori M (2018). Robust real-time musculoskeletal modeling driven by electromyograms. IEEE Transactions on Biomedical Engineering.

[10] Ma C, Lin C, Williams O, Xu L, Li G (2020). Continuous estimation of upper limb joint angle from semg signals based on sca-lstm deep learning approach. Biomedical Signal Processing and Control.

[11] Atzori, M., Cognolato, M. & Müller, H. Deep learning with convolutional neural networks applied to electromyography data: A resource for the classification of movements for prosthetic hands. *Frontiers in Neurorobotics* **10**, 10.3389/fnbot.2016.00009 (2016). 10.3389/fnbot.2016.00009 PMC5013051 27656140

[12] Liu, G. *et al*. semg-based continuous estimation of knee joint angle using deep learning with convolutional neural network. In *2019 IEEE 15th International Conference on Automation Science and Engineering (CASE)*, 140–145, 10.1109/COASE.2019.8843168 (2019).

[13] Zhang Q, Fragnito N, Bao X, Sharma N (2022). A deep learning method to predict ankle joint moment during walking at different speeds with ultrasound imaging: A framework for assistive devices control. Wearable Technologies.

[14] Zhang Q, Clark Q, Franz J, Sharma N (2022). Personalized fusion of ultrasound and electromyography-derived neuromuscular features increases prediction accuracy of ankle moment during plantarflexion. Biomedical Signal Processing and Control.

[15] Bitzer, S. & van der Smagt, P. Learning emg control of a robotic hand: towards active prostheses. In *Proceedings 2006 IEEE International Conference on Robotics and Automation, 2006. ICRA 2006*., 2819–2823, 10.1109/ROBOT.2006.1642128 (2006).

[16] Saxby D (2020). Machine learning methods to support personalized neuromusculoskeletal modelling. Biomech Model Mechanobiol.

[17] Schreiber, C. & Moissenet, F. A multimodal dataset of human gait at different walking speeds established on injury-free adult participants. *Scientific Data* **6**(111) (2019). 10.1038/s41597-019-0124-4 PMC6610108 31270327

[18] Lencioni, T., Carpinella, I., Rabuffetti, M., Marzegan, A. & Ferrarin, M. Human kinematic, kinetic and emg data during different walking and stair ascending and descending tasks. *Scientific Data* **6**(309) (2019). 10.1038/s41597-019-0323-z PMC6897988 31811148

[19] Moreira, L., Figueiredo, J., Fonseca, P., Vilas-Boas, J. & Santos, C. Lower limb kinematic, kinetic, and emg data from young healthy humans during walking at controlled speeds. *Scientific Data* **8**(103) (2021). 10.1038/s41597-021-00881-3 PMC8041842 33846357

[20] Zhang, Q. Experimental data of semg, us imaging, grf, and markers for walking on treadmill across multiple speeds. *IEEE Dataport* (2022).

[21] Reznick, E. *et al*. Lower-limb kinematics and kinetics during continuously varying human locomotion. *Scientific Data* **8**(282) (2021). 10.1038/s41597-021-01057-9 PMC8553836 34711856

[22] Matran-Fernandez, A., Rodríguez Martínez, I., Poli, R., Cipriani, C. & Citi, L. Seeds, simultaneous recordings of high-density emg and finger joint angles during multiple hand movements. *Scientific Data* **6**(186) (2019). 10.1038/s41597-019-0200-9 PMC6768861 31570723

[23] Jarque-Bou, N., Vergara, M., Sancho-Bru, J., Gracias-Ibáñez, V. & Roda-Sales, A. A calibrated database of kinematics and emg of the forearm and hand during activities of daily living. *Scientific Data* **6**(270) (2019). 10.1038/s41597-019-0285-1 PMC6848200 31712685

[24] Maleševic, N. *et al*. A database of high-density surface electromyogram signals comprising 65 isometric hand gestures. *Scientific Data* **8**(63) (2021). 10.1038/s41597-021-00843-9 PMC7892548 33602931

[25] Furmanek, M., Mangalam, M., Yarossi, M., Lockwood, K. & Tunik, E. A kinematic and emg dataset of online adjustment of reachto- grasp movements to visual perturbations. *Scientific Data* **9**(23) (2022). 10.1038/s41597-021-01107-2 PMC8782875 35064126

[26] Rojas-Martínez, M. *et al*. High-density surface electromyography signals during isometric contractions of elbow muscles of healthy humans. *Scientific Data* **7**(397) (2020). 10.1038/s41597-020-00717-6 PMC7670452 33199696

[27] Pagliari D, Pinto L (2015). Calibration of kinect for xbox one and comparison between the two generations of microsoft sensors. Sensors.

[28] Hernández, Ó. G. OHernandezr/Manipulability (2021).

[29] Hernández ÓG (2022). Science Data Bank.

[30] Yoshikawa T (1985). Manipulability of robotic mechanisms. The International Journal of Robotics Research.

[31] Yoshikawa, T. Dynamic manipulability of robot manipulators. In *Proceedings. 1985 IEEE International Conference on Robotics and Automation*, vol. 2, 1033–1038, 10.1109/ROBOT.1985.1087277 (Institute of Electrical and Electronics Engineers, 1985).

[32] Denavit J, Hartenberg RS (1965). A kinematic notation for lower-pair mechanisms based on matrices. Journal of Applied Mechanics.

[33] Corke, P. *Robotics, Vision and Control - Fundamental Algorithms in MATLAB®*, vol. 73 of *Springer Tracts in Advanced Robotics* (Springer, 2011).

[34] Kucuk S, Bingul Z (2006). Comparative study of performance indices for fundamental robot manipulators. Robotics and Autonomous Systems.

[35] Ke, G. *et al*. Lightgbm: A highly efficient gradient boosting decision tree. In Guyon, I. *et al*. (eds.) *Advances in Neural Information Processing Systems*, vol. 30 (Curran Associates, Inc., 2017).

[36] McCarty, D., Kim, H. & Lee, H. Evaluation of light gradient boosted machine learning technique in large scale land use and land cover classification. *Environments* **7**(10), 10.3390/environments7100084 (2020).

[37] Sheridan, R., Liaw, A. & Tudor, M. Light gradient boosting machine as a regression method for quantitative structure-activity relationships. https://lightgbm.readthedocs.io/. Accessed: 2023-02-1630.

[38] Khiari, J. & Olaverri-Monreal, C. Boosting algorithms for delivery time prediction in transportation logistics. *2020 International Conference on Data Mining Workshops (ICDMW)*10.1109/icdmw51313.2020.00043 (2020).

[39] Friedman JH (2001). Greedy function approximation: A gradient boosting machine. The Annals of Statistics.

